# Coverage Analysis of 5G Intelligent High-Speed Railway System Based on Beamwidth-Adaptive Free-Space Optical Communication

**DOI:** 10.3390/s25164906

**Published:** 2025-08-08

**Authors:** Shuai Dong, Zhi-Zhao Zeng, Dan-Ting Zhang, Zi-Qi Sun, Jin-Yuan Wang

**Affiliations:** 1 School of Communications and Information Engineering, Nanjing University of Posts and Telecommunications, Nanjing 210003, China; 1223014125@njupt.edu.cn (S.D.); 1223014126@njupt.edu.cn (Z.-Z.Z.); 1223014127@njupt.edu.cn (D.-T.Z.); 1223014128@njupt.edu.cn (Z.-Q.S.); 2 Chuan and Zang Smart Tourism Engineering Research Center of Colleges and Universities of Sichuan Province, Sichuan Tourism University, Chengdu 610100, China

**Keywords:** high-speed railway, free-space optical communication, beamwidth-adaptive, narrow-strip-shaped cell, edge coverage probability, percentage of cell coverage area

## Abstract

The rapid development of intelligent high-speed railways (HSRs) has significantly improved the transportation efficiency of modern transit systems, while also imposing higher bandwidth demands on mobile communication systems. Free-space optical (FSO) communication technology, as a promising solution, can effectively meet the high-speed data transmission requirements in intelligent HSR scenarios. In this paper, we consider an intelligent HSR system based on beamwidth-adaptive FSO communication and investigate the coverage performance of the system. Different from the circular cells used in traditional radio frequency wireless communication systems, this paper focuses on the coverage problem of narrow-strip-shaped cells in HSR systems based on FSO communication. When the transmitter emits a wide beam, the channel gain includes geometric loss, atmospheric attenuation, and atmospheric turbulence. When the transmitter emits a narrow beam, the channel gain includes pointing error, atmospheric attenuation, and atmospheric turbulence. To adapt the width of the transmitter’s beam, we propose a beamwidth-adaptive HSR system and a beamwidth-adaptive method. Furthermore, we derive closed-form expressions of the edge coverage probability (ECP) and the percentage of cell coverage area (CCA), where the ECP is the probability that the received signal-to-noise ratio at the cell edge is greater than or equal to a given threshold, and the percentage of CCA dictates the percentage of locations within a cell that are not in outage. The accuracy of the derived theoretical expressions is validated through Monte-Carlo simulations. The average relative error of the ECP between theoretical and simulation results is only 0.035%, and the corresponding error of the percentage of CCA is 0.087%. In addition, the impacts of factors such as cell diameter, transmission power, signal-to-noise ratio threshold, and weather visibility on coverage performance are also discussed.

## 1. Introduction

In modern transportation systems, with the acceleration of urbanization and the increasing demand for convenient and efficient travel, high-speed railways (HSRs) have rapidly developed worldwide as a key mode of transportation. The existing global system for mobile communication-railway (GSM-R) is a narrow-band communication system with low bandwidth and data rate, which cannot meet the requirements of intelligent railway services. Long term evolution-railway (LTE-R) was proposed for railway communication system evolution at the beginning of 2010s. However, it cannot meet the requirements of ultra-reliability and high coverage for future railways. To satisfy large data rate and high coverage communication, and support intelligent application scenarios, the fifth generation-railway (5G-R) has drawn much attention in recent years. Currently, most 5G intelligent HSR systems typically rely on radio frequency (RF) wireless communication technologies to provide internet access services for passengers [1,2,3]. However, with the continuous advancement of railway operations, 5G intelligent HSR communication systems face challenges such as transmission rate bottlenecks and degradation of service quality (such as coverage quality) due to limitations in radio wave bandwidth and spectrum interference [4,5]. Consequently, exploring efficient and reliable communication solutions has become crucial for enhancing the service quality of HSRs.

Free-space optical (FSO) communication technology, owing to its advantages of high bandwidth [6,7], license-free operation [8], and excellent anti-interference capability, has gradually become an ideal choice to address HSR communication issues and shows great potential for enabling high-speed communications within HSR environments. For example, in response to the demand for high data rate transmission in HSRs, prior research has proposed an FSO system equipped with a fast handover mechanism [9].

Atmospheric turbulence and atmospheric attenuation caused by aerosol scattering are key challenges for laser transmission in FSO communications. Studies have shown that turbulence causes random wavefront distortions, leading to beam spreading, wandering, and intensity scintillation, which seriously degrade link reliability [10,11]. Meanwhile, aerosol particles in the atmosphere attenuate and perturb the optical signal through absorption and scattering, especially under low-visibility conditions such as fog and haze, where their impact becomes more pronounced [12,13]. These effects not only limit the available frequency bands and stability of laser communication links but also impose higher demands on beam focusing and transmission strategies.

In wireless communications, cell coverage analysis is an essential part of system design, aimed at ensuring reliable communication services for users in various geographical locations. Evaluating the coverage performance of a communication systems involves studying multiple metrics, such as the percentage of cell coverage area (CCA) and edge coverage probability (ECP) [14,15]. Although cell coverage performance analysis of HSR systems based on RF communications has been extensively studied [16,17], coverage analysis for HSR systems based on FSO communication remains insufficient. Considering HSR systems based on FSO communications as a promising alternative gradually compensating traditional RF-based HSR systems, an in-depth analysis of their cell coverage performance is imperative to provide theoretical support for subsequent system optimization.

**Figure 1:**
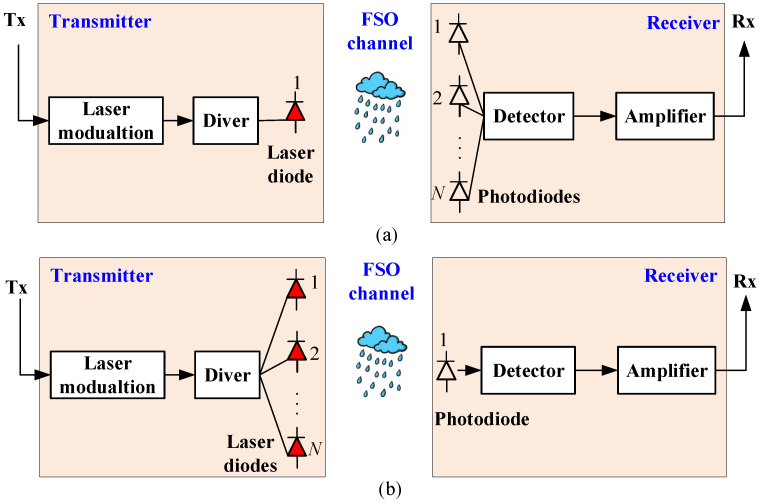
Block diagram of FSO communication systems. (**a**) A single-input-multiple-output FSO system; (**b**) A multiple-input-single-output FSO system.

The typical link distance in FSO systems varies with the application scenario, but it is generally kept between several hundred meters and a few kilometers [18] to strike a balance between signal quality and practical deployment constraints. To maintain continuous FSO links between running trains and ground stations, railway operators need to deploy a large number of FSO base stations (BSs) to achieve seamless coverage. Additionally, during handovers between coverage areas of different BSs, communication interruptions and significant handover delays may occur. To address this issue, related studies have proposed architectures that install multiple receivers on the train to minimize the number of handovers and reduce interruption time [19], as shown in Figure 1a. Other research has suggested deploying multiple transmitters to extend the transmission range of FSO links [20], as shown in Figure 1b. However, increasing the number of transmitters or receivers incurs high costs and increases system complexity. To reduce system cost and complexity, this paper considers a single receiver that is installed on the roof of the train’s front car. This setup has been widely adopted in the literature [17,21,22,23] and ensures an unobstructed line-of-sight to the base stations and effectively prevents signal interference from ground obstacles. Moreover, with minimal vibration at the front car, this location is ideal for maintenance and does not encroach upon passenger space, confirming it as a preferred installation location.

Moreover, weather conditions have strong effects on the performance of FSO communication systems. In [24], a robust optimization approach was proposed for the design of FSO communication networks. Different weather conditions were modeled based on meteorological records for a given time period to estimate the corresponding link failure ratios. Considering various weather conditions, the beam width was optimized for FSO communication links [25]. Since the single-beam FSO system is vulnerable to atmospheric attenuation, a multi-beam FSO system was designed and analyzed to produce the best system capable of tackling the effect of tropical weather [26]. Different from [24,25,26], we consider different visibility distances to characterize the weather conditions. As shown in (6)–(8), it can be observed that different weather conditions have different visibility distances, and thus have different attenuation coefficients.

In FSO communications, transmit beams can be categorized into narrow beams and wide beams. Most studies consider laser beams with a divergence angle of less than 0.0057° as narrow beams, while those exceeding 0.0057° are classified as wide beams [27,28,29]. However, current definitions remain qualitative, lacking a practical method to determine beam width in real-world applications, and the beam width directly affects whether or not the receiver can stably capture the signal. Since the line-of-sight of FSO links is susceptible to train motion, track irregularities, and atmospheric turbulence, narrow-beam transmission leads to significant pointing/tracking errors [30,31], rendering the receiver unable to stably receive the signal. Therefore, for the transmission of narrow beams, a complex and precise alignment system is required. Traditional methods usually employ an acquisition-tracking-pointing (ATP) module [7,9,32,33] to ensure accurate alignment between the transmitter and the receiver. However, this cannot entirely eliminate the effects of pointing errors. In contrast, when the transmitter emits a wide beam, it generates a larger light spot at the receiver, fully covering the receiver and eliminating the influence of pointing error [21,34]. In this case, the receiver can stably receive the signal, and the system does not require an ATP module [21,35]. However, the energy divergence characteristic of the wide beam introduces significant geometric losses, which affects system performance. Currently, existing systems generally adopt a fixed ATP scheme and fail to make adaptive adjustments according to actual beam characteristics. As a result, the system either incurs unnecessary energy consumption or suffers from poor reception performance. Therefore, it is necessary to design a method that enables the receiver to determine whether the transmitter’s beamwidth is wide or narrow based on the received signal strength, thereby adaptively adjusting the activation state of the ATP module.

This paper considers an intelligent HSR system based on beamwidth-adaptive FSO communication. Although existing works have considered adaptive interleaver [36] and adaptive combining techniques [37,38] for FSO communications, they are different from the beamwidth-adaptive solution considered in this paper. We establish a system model that accounts for both wide-beam and narrow-beam transmissions and propose a beamwidth-adaptive method to enhance the system efficiency. In addition, we further study the cell coverage performance in the HSR system based on beamwidth-adaptive FSO communication with narrow-strip-shaped cells and the hard handover scheme. The key contributions of this work are summarized as follows:

- We construct a model of an intelligent HSR system based on beamwidth-adaptive FSO communication with narrow-strip-shaped cells. In this model, the cell diameter and cell edge are defined, and two scenarios of transmitter beamwidth (i.e., wide beam and narrow beam) are considered. When the transmitter emits a wide beam, the channel gain includes geometric loss, atmospheric attenuation, and atmospheric turbulence. When the transmitter emits a narrow beam, the channel gain includes pointing error, atmospheric attenuation, and atmospheric turbulence.
- We propose a beamwidth-adaptive FSO communication system. By incorporating an additional transmitter controller and infrared emitter into the transmitter unit, as well as an additional receiver controller and infrared receiver into the receiver unit, the system is capable of adaptively managing the on or off state of the ATP module.
- We propose a beamwidth-adaptive method. During the handover process, the receiver controller assesses the beam width of the target transmitter by detecting the signal strength received from the target transmitter. In conjunction with the infrared emitter, infrared receiver, and transmitter controller, it adaptively controls the on or off state of the ATP modules in both the transmitter and receiver, thereby achieving dynamic alignment and tracking of the optical beams. Specifically, when the target transmitter emits a wide beam, the system keeps the ATP module off to conserve energy; when a narrow beam is transmitted, the ATP module is activated to ensure precise beam alignment between the transmitter and receiver.
- We analyze the coverage performance at the cell edge by studying the statistical characteristics of the received signal-to-noise ratio (SNR). Based on the definition of ECP and utilizing integral formulas of the Meijer’s G-function, closed-form expressions of ECP are derived for both the wide-beam and narrow-beam cases.
- We present the average coverage performance of the cells, which is characterized by the percentage of CCA. The closed-form expressions of the percentage of CCA for the cases of wide beams and narrow beams are derived by utilizing the mathematical definition, the integral formula of the Meijer’s G-function, and the composite trapezoidal integration approximation method.
- Numerical results are presented, showing good agreement between theoretical and simulation results, thereby validating the accuracy of the derived expressions. In addition, the effects of cell diameter, transmit power, SNR threshold, and weather visibility on coverage performance are analyzed.

The remaining parts of this paper are organized as follows. Section 2 introduces the system and channel models for the HSR system based on the beamwidth-adaptive FSO communication. Section 3 presents the beamwidth-adaptive FSO system and method. Section 4 analyzes the theoretical expressions of the ECP and the percentage of CCA for both the wide-beam system and the narrow-beam system. The numerical results are presented in Section 5. Finally, conclusions are drawn in Section 6.

## 2. System and Channel Models

### 2.1. System Model

**Figure 2:**
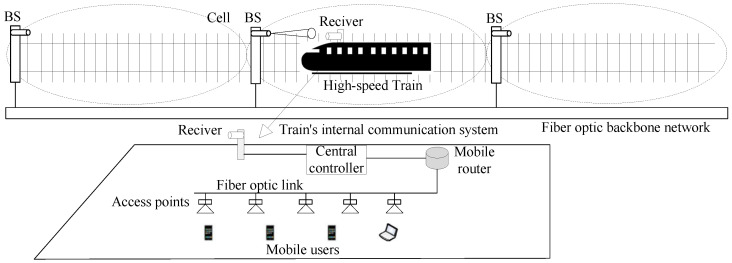
An HSR system based on beamwidth-adaptive FSO communication with narrow-strip-shaped cells.

As shown in Figure 2, we consider an HSR system based on beam-adaptive FSO communication, where multiple BSs are uniformly deployed along the railway track. Each BS is equipped with a transmitter for signal transmission and connected to the optical fiber backbone network for information exchange with service providers. The receiver captures the optical signal from the transmitter via the FSO channel and immediately forwards it to the train’s internal communication system. In this system, only one BS is activated and enters operational mode exclusively when a receiver enters its coverage area, while all other BSs remain in energy-saving sleep mode. The train’s internal communication system includes fiber optic links, a central controller, a mobile router, on-board access points, and mobile users. On the train, mobile users first connect to the on-board access points, which are connected to the mobile router via fiber links. The central controller is responsible for managing the system to optimize network performance. Therefore, the entire communication link is divided into two parts: the backhaul link (i.e., the BS-receiver link) and the front-end link (i.e., the AP-user link). Since the front-end link communication is similar to widely studied cellular networks, the main challenge for HSR communication lies in the backhaul link. Thus, this paper focuses only on the backhaul link. For reasons that are naturally associated with traffic characteristics, this paper considers the FSO communication-based downlink transmission only. For uplink transmission, another type of communication (such as WiFi, Li-Fi, 5G, etc.) can be used to avoid interference.

**Figure 3:**
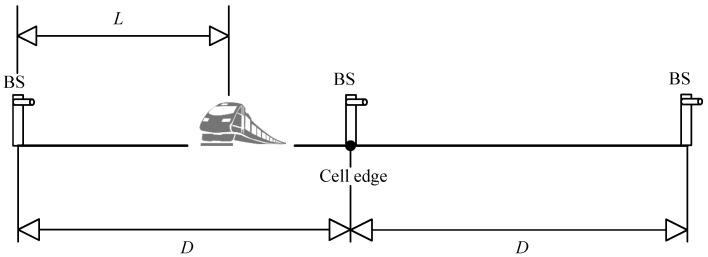
Cell coverage scenario.

Due to the good directionality of laser signals, narrow-strip-shaped cells are considered. Figure 3 illustrates the cell coverage scenario. Since BSs are deployed near the track, the vertical distance between each BS and the track can be neglected. The transmitter’s height above the ground is slightly higher than that of the receiver, which can be approximated as the transmitter emitting laser signals horizontally to the receiver. Therefore, the distance between each transmitter and the receiver can be considered as the horizontal distance between these two nodes. Each transmitter covers a certain range, with a cell diameter *D*. To facilitate the analysis, we assume that the distance between the receiver and the currently communicating transmitter is denoted as *L*, which satisfies 0≤L≤D.

In this paper, we consider the intensity modulation and direct detection channel with on–off keying (OOK) modulation, which is widely used in many systems. The transmitter modulates data onto the instantaneous intensity of the optical beam. The electrical signal received at the receiver can be expressed as

(1) y=Rhx+n

where $x\in \{0,2P_{\mathrm{tx}}\}$ represents the optical signal by the transmitter, and it is selected from the OOK constellation with equal probability for symbols 0 and $2P_{\mathrm{tx}}$. $P_{\mathrm{tx}}$ denotes the average transmitted optical power. *R* denotes the photoelectric conversion efficiency. *n* is the additive white Gaussian noise with mean zero and variance $\sigma_{n}^{2}$. *h* represents the channel gain. When the transmitter emits a wide beam, the channel gain includes geometric loss [21], atmospheric attenuation, and atmospheric turbulence. Conversely, when the transmitter emits a narrow beam, the channel gain includes pointing error [39], atmospheric attenuation, and atmospheric turbulence. Therefore, *h* in (1) is given by [21,39]

(2) $$h=\left\{\begin{array}{c} \begin{array}{cc} h_{g}h_{l}h_{a} & \mathrm{Wide}\;\mathrm{beam} \end{array}\\ \begin{array}{cc} h_{l}h_{a}h_{p} & \mathrm{Narrow}\;\mathrm{beam} \end{array} \end{array}\right.$$

where $h_{g}$ represents the geometric loss of optical power, $h_{l}$ is the atmospheric attenuation, $h_{a}$ is the atmospheric turbulence, and $h_{p}$ is the pointing error.

The received electrical power can be expressed as

(3) $$P_{\mathrm{rx}}\left(L\right)=2P_{\mathrm{tx}}^{2}R^{2}h^{2}$$

Then, the received electrical SNR can be expressed as

(4) $$SNR\left(L\right)=\frac{P_{\mathrm{rx}}\left(L\right)}{\sigma_{n}^{2}}=\left\{\begin{array}{c} \begin{array}{cc} \frac{2P_{\mathrm{tx}}^{2}R^{2}}{\sigma_{n}^{2}}{\left(h_{g}h_{l}h_{a}\right)}^{2} & \mathrm{Wide}\;\mathrm{beam} \end{array}\\ \begin{array}{cc} \frac{2P_{\mathrm{tx}}^{2}R^{2}}{\sigma_{n}^{2}}{\left(h_{l}h_{a}h_{p}\right)}^{2} & \;\mathrm{Narrow}\;\mathrm{beam} \end{array} \end{array}\right.$$

### 2.2. Channel Model

#### 2.2.1. Geometric Loss

Geometric loss is the loss generated due to the diffusion of the transmitted optical beam between the transmitter and the receiver. Generally, the optical beam spreads to a size larger than the receiving aperture, and the energy of this “overfilling” is lost. The geometric loss $h_{g}$ in (2) can be expressed as [21]

(5) $$h_{g}=\frac{B^{2}}{\theta^{2}L^{2}},\;\;\;0<h_{g}\leq 1$$

where *B* is the diameter of the receiver, θ is the divergence angle of the transmitter, and *L* is the communication distance between the transmitter and the receiver. When θ is large, the geometric loss is less than one, and its effect on system performance cannot be ignored.

#### 2.2.2. Atmospheric Attenuation

When the optical beam passes through the atmosphere, power attenuation occurs due to absorption and scattering. Absorption is primarily caused by water vapor and carbon dioxide, while scattering is caused by molecules, dust particles, and water droplets in fog, rain, or snow. There are two kinds of scattering: Mie scattering and Rayleigh scattering. In the FSO waveband, Mie scattering is the main cause of attenuation, while Rayleigh scattering is negligible [40]. The results show that the influence of Mie scattering on the power attenuation of the optical beam is much greater than that of absorption and Rayleigh scattering. Especially when the diameter of the particles is proportional to the wavelength of the laser beam, the generated Mie scattering impact is sufficiently high. The radius of fog particles is generally 1–20 nm, and the radius of haze particles is generally 0.01–1 nm [21]. Therefore, fog and haze are composed of small particles, and their sizes are close to the wavelength of the optical beam. This is why the most unfavorable environmental conditions for FSO communications are fog and haze. In contrast, due to the large particle size (about 0.1–5 mm), rainfall has a small impact on FSO communication. Moreover, the lens in the system will continuously be covered with dust (and sometimes dirt and snow), which will also result in atmospheric attenuation. To eliminate this influence, three methods, i.e., mechanical or manual lens wiping method, spray lens cleaning method, and lens heating antifreeze method, can be employed. Due to the effects of absorption, scattering, fog, haze, and rainfall, the atmospheric attenuation $h_{l}$ in (2) can be expressed as [30]

(6) $$h_{l}=e^{-\gamma \left(\lambda \right)L}$$

where γ(λ) denotes the attenuation coefficient, and it can be expressed as

(7) $$\gamma \left(\lambda \right)=\frac{17.35}{V}{\left(\frac{\lambda }{550}\right)}^{-q}$$

where *V* (in km) is the visibility distance and λ (in m) is the wavelength of the laser beam. Moreover, *q* is the size distribution of scattering particles under different weather conditions, which can be expressed as [30]

(8) $$q=\left\{\begin{array}{ll} 1.6, & \mathrm{if}\;V>50\;\mathrm{km}\\ 1.3, & \mathrm{if}\;6\;\mathrm{km}<V<50\;\mathrm{km}\\ 0.16V+34, & \mathrm{if}\;1\;\mathrm{km}<V<6\;\mathrm{km}\\ V-0.5, & \mathrm{if}\;0.5\;\mathrm{km}<V<1\;\;\mathrm{km}\\ 0, & \mathrm{if}\;V<0.5\;\mathrm{km} \end{array}\right.$$

Therefore, the atmospheric attenuation under different weather conditions can be calculated using Equations (6)–(8).

#### 2.2.3. Atmospheric Turbulence

In the past two decades, many statistical models of the intensity fluctuations of the FSO channel have been proposed. For weak turbulence, the probability density function (PDF) of the intensity fluctuations is modeled as a log-normal distribution. In the strong turbulence region, the log-normal distribution cannot characterize the scintillation effect, and the Gamma–Gamma distribution is used to model the atmospheric fading. A statistical model called the Málaga or M distribution has been proposed [39], which is suitable for the irradiance fluctuations of unbounded optical wavefronts propagating through a turbulent medium under all irradiance fluctuation conditions in homogeneous and isotropic turbulence. Compared with experimental data and existing statistical models, the M distribution has been verified. This distribution unifies all the previously proposed models into a single model, which is valid for each turbulence condition. Therefore, it is a more general turbulence model. Mathematically, the PDF of $h_{a}$ in (2) can be expressed as [39]

(9) $$f_{h_{a}}\left(h_{a}\right)=A\sum_{k=1}^{\beta }a_{k}{h_{a}}^{\frac{\alpha +k}{2}-1}K_{\alpha -k}\left(2\sqrt{\frac{\alpha \beta h_{a}}{\xi_{g}\beta +\Omega^{\prime }}}\right)$$

where *A* and $a_{k}$ can be expressed as

(10) $$\left\{\begin{array}{c} A\overset{∆}{=}\frac{2\alpha^{\frac{\alpha }{2}}}{{\xi_{g}}^{1+\frac{\alpha }{2}}\Gamma \left(\alpha \right)}{\left(\frac{\xi_{g}\beta }{\xi_{g}}\beta +\Omega^{\prime }\right)}^{\beta +\frac{\alpha }{2}}\\ a_{k}\overset{∆}{=}\left(\begin{array}{c} \beta -1\\ k-1 \end{array}\right){\left(\frac{\xi_{g}\beta +\Omega^{\prime }}{\Gamma (k)}\right)}^{1-\frac{k}{2}}{\left(\frac{\Omega^{\prime }}{\xi_{g}}\right)}^{k-1}{\left(\frac{\alpha }{\beta }\right)}^{\frac{k}{2}} \end{array}\right.$$

where α is a positive parameter related to the effective number of large-scale cells in the scattering process, $\xi_{g}$ is the average optical power of the classical scattering component received by the off-axis eddy current, β is a natural number representing the fading parameter quantity, the parameter $\Omega^{\prime }$ represents the average optical power of the coherent contribution, Γ(k) represents the Gamma function, and $K_{v}\left(\cdot \right)$ is the modified Bessel function of the second kind with order *v*.

#### 2.2.4. Pointing Error

For a line-of-sight link in FSO communication, the pointing accuracy is an important factor determining the link performance and reliability. However, vibration and swaying of the moving train will cause pointing error and signal fading at the receiver. The PDF of $h_{p}$ in (2) can be expressed as [39]

(11) $$f_{h_{p}}\left(h_{p}\right)=\frac{r^{2}}{A_{0}^{r^{2}}}h_{p}^{r^{2}-1},\;\;\;\;0\leq h_{p}\leq A_{0}$$

where *r* is the ratio of the equivalent beam radius at the receiver to the standard deviation of the pointing error displacement at the receiver. $A_{0}={\left[\mathrm{erf}\left(\sqrt{\pi }B/2\sqrt{2}w_{L}\right)\right]}^{2}$, $w_{L}=w_{0}\sqrt{1+{\left[\lambda L/\left(\pi w_{0}^{2}\right)\right]}^{2}}$ is the beam waist at a distance *L* (the radius calculated at $e^{-2}$), $w_{0}$ is the beam waist radius, and erf(·) is the error function.

## 3. Proposed Beamwidth-Adaptive FSO Communication System and Method

In this section, we present a beamwidth-adaptive FSO communication system to cope with the emitted beamwidth of the transmitter. First, we introduce the system architecture, followed by the beamwidth-adaptive method.

### 3.1. Beamwidth-Adaptive System

**Figure 4:**
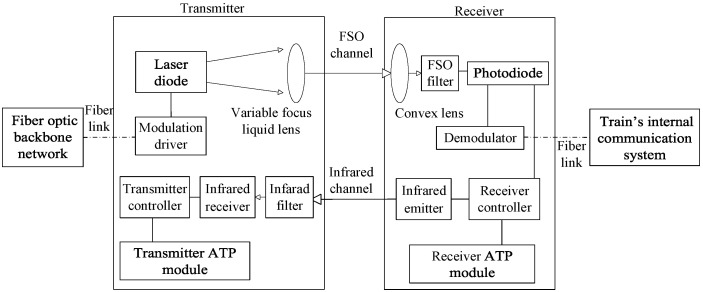
The structure of the beamwidth-adaptive FSO communication system.

Figure 4 illustrates the proposed structure of the beamwidth-adaptive FSO communication system. The transmitter includes a laser diode, a variable focus liquid lens, a modulation driver, a transmitter controller, an infrared filter, an infrared receiver, and a transmitter ATP module. The modulator driver serves as a critical interface for the input signal from the optical fiber backbone network. It employs intensity modulation to superimpose the baseband electrical signal onto the optical carrier, thereby facilitating the transmission of information. Subsequently, the laser diode, driven by the modulated signal, efficiently converts the electrical signal into an optical signal, ultimately generating an optical signal that carries the desired information. We assume that the laser diode emits a collimated beam. The variable focus liquid lens [33] dynamically adjusts the curvature of its liquid-filled membrane via an electromagnetic actuator, thereby continuously varying the focal length and tuning the divergence angle of the emitted beam. Then, the transmitter can emit either a wide beam or a narrow beam. The transmitter controller is connected to both the transmitter ATP module and the infrared receiver. It activates the transmitter ATP module when the infrared receiver detects a signal from the infrared transmitter and deactivates the transmitter ATP module when no such infrared signal is detected.

The receiver consists of a convex lens, an FSO filter, a photodiode, a demodulator, a receiver controller, an infrared transmitter, and a receiver ATP module. The convex lens collects the incoming optical signal and focuses it onto the photodiode, which converts the optical signal into an electrical signal. The demodulator decodes the signal into its original form, which is then transmitted via the optical fiber link to the train’s internal communication system. The receiver controller is interconnected with the photodiode, the receiver ATP module, and the infrared transmitter. It is designed to assess the received signal strength and, based on this assessment, determine whether or not to activate the receiver ATP module and the infrared emitter during the handover process. Additionally, when both the transmitter ATP module and receiver ATP module are activated simultaneously, they enable mutual beam tracking between the transmitter and receiver. Note that the FSO filter and the infrared filter are employed in the system to suppress the influence of background illumination.

### 3.2. Beamwidth-Adaptive Method

In the system, the transmitters’ states are divided into three categories: the transmitter currently maintaining communication with the receiver is referred to as the source transmitter; the transmitter that is about to communicate with the receiver is called the target transmitter; and the remaining transmitters that have no communication with the receiver are silent transmitters.

**Figure 5:**
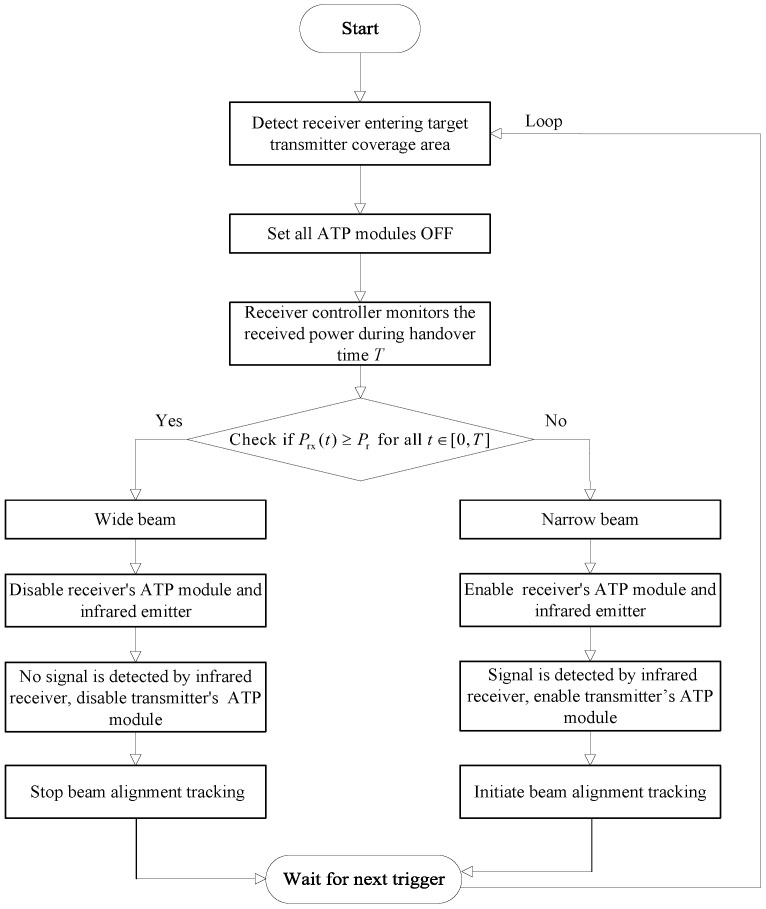
Flow of the beamwidth-adaptive method.

The flow of the beamwidth-adaptive method is illustrated in Figure 5. When the receiver leaves the communication coverage area of the source transmitter and enters that of the target transmitter, a handover occurs. Initially, all transmitters’ ATP modules and the receiver’s ATP module are set to off at the start of the handover. To facilitate the descriptions, we let *T* be the handover interval and $P_{\mathrm{th}}$ be the received power threshold. Based on the characteristics of wide and narrow beams, the receiver can reliably receive continuous power when a wide beam is transmitted, thereby maintaining the received power above a predetermined threshold. In contrast, when transmitting a narrow beam, pointing errors often cause the received power to fluctuate, making it difficult to consistently stay above the threshold. Therefore, at the time interval *T*, if the received power $P_{\mathrm{rx}}$ from the target transmitter signal remains consistently greater than or equal to $P_{\mathrm{th}}$, the system judges that the target transmitter is emitting a wide beam; otherwise, it is judged to be emitting a narrow beam. This decision method can be expressed as

(12) $$\left\{\begin{array}{cc} \mathrm{Wide}\;\mathrm{beam} & \begin{array}{cc} \mathrm{if} & P_{\mathrm{rx}}\left(t\right)\geq P_{\mathrm{th}},\forall t\in \left[0,T\right] \end{array}\\ \mathrm{Narrow}\;\mathrm{beam} & \;\;\;\;\mathrm{otherwise}\;\;\;\;\;\;\;\;\;\;\;\;\;\;\;\;\;\;\;\;\;\;\;\;\;\;\;\;\;\;\;\;\;\;\;\;\;\;\;\;\;\;\;\;\;\;\;\;\;\;\;\;\;\;\;\;\;\;\;\;\;\;\;\;\;\;\;\;\;\;\;\;\;\;\; \end{array}\right.$$

When the target transmitter is judged to be emitting a wide beam, the receiver controller keeps the receiver ATP module and infrared emitter turned off. Since the infrared receiver does not detect any signals from the infrared emitter, the transmitter controller keeps the transmitter ATP module off. At this time, both the transmitter and receiver ATP modules are off, and no beam alignment or tracking occurs between the transmitter and receiver.

When the target transmitter is judged to be emitting a narrow beam, the receiver controller turns on the receiver ATP module and the infrared emitter. The infrared emitter sends signals to the infrared the receiver. As a control channel, the infrared channel is used to transmit ATP control signaling. Naturally, this control channel can also be implemented by the RF technique. Meanwhile, the transmitter controller also activates the transmitter ATP module to enable alignment and tracking of the optical beam between the target transmitter and receiver.

Upon completion of the aforementioned operations, the system transitions into a “wait for next trigger” state, awaiting the detection signal indicating that the receiver has entered the coverage area of the target transmitter once again. At this juncture, the process loops back to the detection phase, thereby enabling continuous monitoring of the receiver’s status and facilitating adaptive adjustments as required. To provide more rigorous details, we provide a pseudocode for the beamwidth-adaptive method, as shown in Algorithm 1.
**Algorithm 1** Beamwidth-adaptive Method1:**if** the receiver enters the coverage area of a target transmitter **then**2:    Set all ATP modules OFF3:    **for** t=0:∆t:T **do** % ∆t is the step size of time4:        Calculate the received electrical power $P_{\mathrm{rx}}\left(t\right)=2P_{\mathrm{tx}}^{2}R^{2}h^{2}$;5:    **end for**6:    **if** there exists $P_{\mathrm{rx}}\left(t\right)<P_{\mathrm{th}}$ **then**7:        The transmitted beam is a narrow beam;8:        The receiver controller turns on the receiver’s ATP module;9:        The infrared emitter transmits an indicator signal;10:      The indicator signal is detected by the infrared receiver;11:      The transmitter controller turns on the transmitter’s ATP module;12:      The beam alignment tracking is implemented.13:  **end if**14:  **if** all received power during the handover period satisfy $P_{\mathrm{rx}}\left(t\right)\geq P_{\mathrm{th}},\forall t$ **then**15:      The transmitted beam is a wide beam;16:      The receiver controller turns off the receiver’s ATP module;17:      The infrared emitter does not transmit any signal to infrared receiver;18:      No indicator signal is detected by the infrared receiver;19:      The transmitter controller keeps the transmitter’s ATP module off;20:      The beam alignment tracking is not implemented.21:  **end if**22:**end if**

## 4. Coverage Performance Analysis

In this section, the coverage performance (such as the ECP and the percentage of CCA) of the HSR system based on beamwidth-adaptive FSO communication is analyzed.

### 4.1. ECP Analysis

In this subsection, the ECP is analyzed. It is well known that ECP is defined as the probability that the SNR at the cell edge (i.e., L=D) exceeds a given threshold $r_{\mathrm{th}}$. When the transmitter emits a wide beam, the ECP is given by

(13) $$\begin{array}{cc} P_{e,1} & =\mathrm{Pr}\left\{\mathrm{SNR}\left(D\right)>r_{\mathrm{th}}\right\}\\ \; & =\mathrm{Pr}\left\{\frac{2P_{tx}^{2}R^{2}}{\sigma_{n}^{2}}{\left(h_{g}h_{l}h_{a}\right)}^{2}>r_{\mathrm{th}}\right\}\\ \; & =\mathrm{Pr}\left\{h_{a}>\gamma_{\mathrm{th}}^{\prime }\right\}\\ \; & =\int_{\gamma_{\mathrm{th}}^{\prime }}^{\infty }f_{h_{a}\left(D\right)}\left(h_{a}\right)dh_{a} \end{array}$$

where $\gamma_{\mathrm{th}}^{\prime }=r_{\mathrm{th}}\sigma_{n}\theta^{2}D^{2}e^{\gamma (\lambda )D}/\left(\sqrt{2}P_{\mathrm{tx}}RB^{2}\right)$.

By solving (13), we obtain the following theorem.

**Theorem** **1.** *For the wide-beam system, the ECP is given by*

(14) $$\begin{array}{cc} P_{e,1} & =1-\frac{A}{2}\sum_{k=1}^{\beta }\left\{a_{k}{\left(\frac{r_{\mathrm{th}}\sigma_{n}\theta^{2}D^{2}e^{\gamma (\lambda )D}}{\sqrt{2}P_{\mathrm{tx}}RB^{2}}\right)}^{\frac{\alpha +k}{2}}\right.\\ \; & \times \left.G_{1,3}^{2,1}\left(\frac{\alpha \beta r_{\mathrm{th}}\sigma_{n}\theta^{2}D^{2}e^{\gamma (\lambda )D}}{\left(\xi_{g}\beta +\Omega^{\prime }\right)\sqrt{2}P_{\mathrm{tx}}RB^{2}}\left|\begin{array}{c} 1-\frac{\alpha +k}{2}\\ \begin{array}{ccc} \frac{\alpha -k}{2} & \frac{k-\alpha }{2} & -\frac{\alpha +k}{2} \end{array} \end{array}\right.\right)\right\} \end{array}$$

*where $G_{m,n}^{p,q}\left(\cdot \right)$ is the Meijer’s G-function.*

**Proof.**  See Appendix A.    □

It is well known that visibility has a significant impact on the coverage performance of FSO communication systems. When considering an ideal weather condition (i.e., V→∞), we obtain the following corollary and reamrk.

**Corollary** **1.** *If the visibility V→∞, the ECP in Theorem 1 reduces to*

(15) $$\begin{array}{cc} P_{e,1} & =1-\frac{A}{2}\sum_{k=1}^{\beta }\left\{a_{k}{\left(\frac{r_{\mathrm{th}}\sigma_{n}\theta^{2}D^{2}}{\sqrt{2}P_{\mathrm{tx}}RB^{2}}\right)}^{\frac{\alpha +k}{2}}\right.\\ \; & \times \left.G_{1,3}^{2,1}\left(\frac{\alpha \beta r_{\mathrm{th}}\sigma_{n}\theta^{2}D^{2}}{\left(\xi_{g}\beta +\Omega^{\prime }\right)\sqrt{2}P_{\mathrm{tx}}RB^{2}}\left|\begin{array}{c} 1-\frac{\alpha +k}{2}\\ \begin{array}{ccc} \frac{\alpha -k}{2} & \frac{k-\alpha }{2} & -\frac{\alpha +k}{2} \end{array} \end{array}\right.\right)\right\} \end{array}$$

**Remark** **1.** *When V→∞, the attenuation coefficient γ(λ) becomes zero, which indicates that the atmospheric attenuation $h_{l}=1$. In other words, under ideal weather conditions, the impact of atmospheric attenuation on the received signal performance can be neglected.*

When the transmitter emits a narrow beam, we can obtain the PDF of *h* as

(16) $$\begin{array}{cc} f_{h}\left(h\right) & =\int f_{h|h_{a}}(h\left|h_{a}\right)f_{h_{a}}\left(h_{a}\right)dh_{a}\\ \; & =\int_{h/\left(h_{l}A_{0}\right)}^{\infty }f_{h_{a}}\left(h_{a}\right)\frac{h}{h_{l}h_{a}}f_{h_{p}}\left(\frac{h}{h_{l}h_{a}}\right)dh_{a} \end{array}$$

where $f_{h|h_{a}}\left(h|h_{a}\right)$ is the conditional PDF of *h* given a turbulence state $h_{a}$. Applying simple random variable transformation on (11) and using (16), we get the PDF of *h* given by [41] (Equation (21))

(17) $$\begin{array}{c} f_{h}\left(h\right)=\frac{r^{2}A}{2h}\sum_{k=1}^{\beta }b_{k}G_{1,3}^{3,0}\left(\frac{\alpha \beta h}{\left(\xi_{g}\beta +\Omega^{\prime }\right)A_{0}e^{-\gamma (\lambda )D}}\;|\;\begin{array}{c} 1+r^{2}\\ r^{2}\;\alpha \;k \end{array}\right) \end{array}$$

where $b_{k}=a_{k}{\left[\alpha \beta /\left(\xi_{g}\beta +\Omega^{\prime }\right)\right]}^{-\left(\alpha +k\right)/2}$.

For the narrow-beam system, the ECP is given by

(18) $$\begin{array}{cc} P_{e,2} & =\mathrm{Pr}\left\{SNR\left(D\right)>r_{\mathrm{th}}\right\}\\ \; & =\mathrm{Pr}\left\{\frac{2P_{\mathrm{tx}}^{2}R^{2}}{\sigma_{n}^{2}}h^{2}>r_{\mathrm{th}}\right\}\\ \; & =\mathrm{Pr}\left\{h>\gamma_{\mathrm{th}}^{\prime \prime }\right\}\\ \; & =\int_{\gamma_{\mathrm{th}}^{\prime \prime }}^{\infty }f_{h(D)}\left(h\right)dh \end{array}$$

where $\gamma_{\mathrm{th}}^{\prime \prime }=\sigma_{n}r_{\mathrm{th}}/\left(\sqrt{2}P_{\mathrm{tx}}R\right)$.

By solving (18), we obtain the following theorem.

**Theorem** **2.** *For the narrow-beam system, the ECP is given by*

(19) $$P_{e,2}=1-\frac{r^{2}A}{2}\sum_{k=1}^{\beta }b_{k}G_{2,4}^{3,1}\left(\frac{\sigma_{n}\alpha \beta r_{\mathrm{th}}e^{\gamma (\lambda )D}}{\left(\xi_{g}\beta +\Omega^{\prime }\right)\sqrt{2}P_{\mathrm{tx}}RA_{0}}\left|\begin{array}{c} \begin{array}{cc} 1 & 1 \end{array}+r^{2}\\ \begin{array}{cccc} r^{2} & \alpha & k & 0 \end{array} \end{array}\right.\right)$$

**Proof.**  See Appendix B.    □

**Corollary** **2.** *If the visibility is V→∞, the ECP in Theorem 2 reduces to*

(20) $$P_{e,2}=1-\frac{r^{2}A}{2}\sum_{k=1}^{\beta }b_{k}G_{2,4}^{3,1}\left(\frac{\sigma_{n}\alpha \beta r_{\mathrm{th}}}{\left(\xi_{g}\beta +\Omega^{\prime }\right)\sqrt{2}P_{\mathrm{tx}}RA_{0}}\left|\begin{array}{c} \begin{array}{cc} 1 & 1 \end{array}+r^{2}\\ \begin{array}{cccc} r^{2} & \alpha & k & 0 \end{array} \end{array}\right.\right)$$

### 4.2. Percentage of CCA Analysis

In this subsection, the percentage of CCA is analyzed. The percentage of CCA is another important metric for coverage analysis and dictates the percentage of locations within a narrow-strip-shaped cell that are not in outage. When the transmitter emits a wide beam, the percentage of CCA is given by

(21) $$\begin{array}{cc} P_{a,1}\; & =\frac{1}{D}\int_{0}^{D}\mathrm{Pr}\{SNR\left(L\right)\geq r_{\mathrm{th}}\}dL\\ \; & =\frac{1}{D}\int_{0}^{D}\int_{\gamma_{\mathrm{th}}^{\prime }}^{\infty }f_{h_{a}}\left(h_{a}\right)dh_{a}dL\\ \; & =\frac{1}{D}\int_{0}^{D}P_{e,1}\left(L\right)dL \end{array}$$

By solving (21), we obtain the following theorem.

**Theorem** **3.** *For the wide-beam system, the percentage of CCA is given by*

(22) $$\begin{array}{cc} P_{a,1} & =\frac{1}{2n}\left\{4-\frac{A}{2}\sum_{k=1}^{\beta }a_{k}\left({\left(\frac{r_{\mathrm{th}}\sigma_{n}\theta^{2}D^{2}e^{\gamma (\lambda )D}}{\sqrt{2}P_{\mathrm{tx}}RB^{2}}\right)}^{\frac{\alpha +k}{2}}\right.\right.\\ \; & \times \;G_{1,3}^{2,1}\left(\frac{\alpha \beta r_{\mathrm{th}}\sigma_{n}\theta^{2}D^{2}e^{\gamma (\lambda )D}}{\left(\xi_{g}\beta +\Omega^{\prime }\right)\sqrt{2}P_{\mathrm{tx}}RB^{2}}\left|\begin{array}{c} 1-\frac{\alpha +k}{2}\\ \frac{\alpha -k}{2},\frac{k-\alpha }{2},-\frac{\alpha +k}{2} \end{array}\right.\right)\\ \; & +\;2\sum_{i=1}^{n-1}{\left(\frac{r_{\mathrm{th}}\sigma_{n}\theta^{2}{\left(\frac{iD}{n}\right)}^{2}e^{\gamma \left(\lambda \right)\frac{iD}{n}}}{\sqrt{2}P_{\mathrm{tx}}RB^{2}}\right)}^{\frac{\alpha +k}{2}}\\ \; & \left.\left.\times \;G_{1,3}^{2,1}\left(\frac{\alpha \beta r_{\mathrm{th}}\sigma_{n}\theta^{2}{\left(\frac{iD}{n}\right)}^{2}e^{\gamma \left(\lambda \right)\frac{iD}{n}}}{\left(\xi_{g}\beta +\Omega^{\prime }\right)\sqrt{2}P_{\mathrm{tx}}RB^{2}}\left|\begin{array}{c} 1-\frac{\alpha +k}{2}\\ \frac{\alpha -k}{2},\frac{k-\alpha }{2},-\frac{\alpha +k}{2} \end{array}\right.\right)\right)\right\} \end{array}$$

*where n represents the number of sub-intervals in the composite trapezoidal integral.*

**Proof.**  See Appendix C.    □

**Corollary** **3.** *If the visibility is V→∞, the percentage of CCA in Theorem 3 reduces to*

(23) $$\begin{array}{cc} P_{a,1} & =\frac{1}{2n}\left\{4-\frac{A}{2}\sum_{k=1}^{\beta }a_{k}\left({\left(\frac{r_{\mathrm{th}}\sigma_{n}\theta^{2}D^{2}}{\sqrt{2}P_{\mathrm{tx}}RB^{2}}\right)}^{\frac{\alpha +k}{2}}\right.\right.\\ \; & \;\times G_{1,3}^{2,1}\left(\frac{\alpha \beta r_{\mathrm{th}}\sigma_{n}\theta^{2}D^{2}}{\left(\xi_{g}\beta +\Omega^{\prime }\right)\sqrt{2}P_{\mathrm{tx}}RB^{2}}\left|\begin{array}{c} 1-\frac{\alpha +k}{2}\\ \frac{\alpha -k}{2},\frac{k-\alpha }{2},-\frac{\alpha +k}{2} \end{array}\right.\right)\\ \; & +\;2\sum_{i=1}^{n-1}{\left(\frac{r_{\mathrm{th}}\sigma_{n}\theta^{2}{\left(\frac{iD}{n}\right)}^{2}}{\sqrt{2}P_{\mathrm{tx}}RB^{2}}\right)}^{\frac{\alpha +k}{2}}\\ \; & \left.\left.\times \;G_{1,3}^{2,1}\left(\frac{\alpha \beta r_{\mathrm{th}}\sigma_{n}\theta^{2}{\left(\frac{iD}{n}\right)}^{2}}{\left(\xi_{g}\beta +\Omega^{\prime }\right)\sqrt{2}P_{\mathrm{tx}}RB^{2}}\left|\begin{array}{c} 1-\frac{\alpha +k}{2}\\ \frac{\alpha -k}{2},\frac{k-\alpha }{2},-\frac{\alpha +k}{2} \end{array}\right.\right)\right)\right\} \end{array}$$

When the transmitter emits a narrow beam, the percentage of CCA is given by

(24) $$\begin{array}{cc} P_{a,2} & =\frac{1}{D}\int_{0}^{D}\mathrm{Pr}\{SNR\left(L\right)\geq r_{\mathrm{th}}\}dL\\ \; & =\frac{1}{D}\int_{0}^{D}\int_{\gamma_{\mathrm{th}}^{\prime \prime }}^{\infty }f_{h(L)}\left(h\right)dhdL\\ \; & =\frac{1}{D}\int_{0}^{D}P_{e,2}\left(L\right)dL \end{array}$$

By solving (24), we obtain the following theorem.

**Theorem** **4.** *For the narrow-beam system, the percentage of CCA is given by*

(25) $$\begin{array}{cc} P_{a,2} & =1-\frac{r^{2}A}{2D\gamma (\lambda )}\sum_{k=1}^{\beta }b_{k}\left\{G_{3,5}^{3,2}\left(\frac{\sigma_{n}\alpha \beta r_{\mathrm{th}}e^{\gamma (\lambda )D}}{\left(\xi_{g}\beta +\Omega^{\prime }\right)\sqrt{2}P_{\mathrm{tx}}RA_{0}}\left|\begin{array}{c} \begin{array}{ccc} 1 & 1 & 1+r^{2} \end{array}\\ \begin{array}{cccc} r^{2} & \alpha & k & \begin{array}{cc} 0 & 0 \end{array} \end{array} \end{array}\right.\right)\right.\\ \; & -\left.G_{3,5}^{3,2}\left(\frac{\sigma_{n}\alpha \beta r_{\mathrm{th}}}{\left(\xi_{g}\beta +\Omega^{\prime }\right)\sqrt{2}P_{\mathrm{tx}}RA_{0}}\left|\begin{array}{c} \begin{array}{ccc} 1 & 1 & 1+r^{2} \end{array}\\ \begin{array}{cccc} r^{2} & \alpha & k & \begin{array}{cc} 0 & 0 \end{array} \end{array} \end{array}\right.\right)\right\} \end{array}$$

**Proof.**  See Appendix D.    □

**Corollary** **4.** *If the visibility is V→∞, the percentage of CCA in Theorem 4 reduces to*

(26) $$\begin{array}{c} P_{a,2}=1-\frac{r^{2}A}{2}\sum_{k=1}^{\beta }b_{k}G_{2,4}^{3,1}\left(\frac{\sigma_{n}\alpha \beta r_{\mathrm{th}}}{\left(\xi_{g}\beta +\Omega^{\prime }\right)\sqrt{2}P_{\mathrm{tx}}RA_{0}}\left|\begin{array}{c} \begin{array}{cc} 1 & 1 \end{array}+r^{2}\\ \begin{array}{cccc} r^{2} & \alpha & k & 0 \end{array} \end{array}\right.\right) \end{array}$$

## 5. Numerical Results

In this section, classical numerical results are presented to validate the cell coverage analysis for a HSR system based on beamwidth-adaptive FSO communications. The main simulation parameters are listed in Table 1. To clarify the simulation process, the pseudocode of the Monte-Carlo simulation is provided in Algorithm 2. As shown in Table 1, the considered system operates at an 850 nm wavelength, which ensures safety and is suitable for deployment in public areas. This wavelength falls within a near-infrared region known for its low risk of adverse effects on human eyes [42]. Moreover, we consider employing lasers classified as Class 1 or Class 1M, which are deemed safe for direct human exposure according to international laser safety standard [43].
**Algorithm 2** Monte-Carlo simulation method1:**Input:** Number of Snapshots *N*, $r_{\mathrm{th}}$, $P_{\mathrm{tx}}$, *R*, $\sigma_{n}$, *D*, other default parameters;2:**Output:** Simulated ECP $P_{a}^{\mathrm{sim}}$ and simulated percentage of CCA $P_{e}^{\mathrm{sim}}$;3:**Initialization:** $P_{\mathrm{ac}}=0$, $P_{\mathrm{sce}}=0$, $N=10^{5}$;4:**for** j=1:N **do**5:    **for** L=0:D/∆:D **do**   %∆ is the number of discrete distances6:        **if** the beam is wide **then**7:           Generate $h_{g}$ and $h_{a}$ according to (5) and (9);8:           Calculate $h_{j}\left(L\right)=h_{g}h_{l}h_{a}$;9:        **else**10:         Generate $h_{a}$ and $h_{p}$ according to (9) and (11);11:         Calculate $h_{j}\left(L\right)=h_{l}h_{a}h_{p}$;12:       **end if**13:       Compute ${\mathrm{SNR}}_{j}\left(L\right)$ at distance *L* according to (4);14:       **if** ${\mathrm{SNR}}_{j}\left(L\right)\geq r_{\mathrm{th}}$ **then**15:         $P_{\sec}=P_{\sec}+1$;16:       **end if**17:   **end for**18:   Compute ${\mathrm{SNR}}_{j}\left(D\right)$ at cell edge according to (4);19:   **if** ${\mathrm{SNR}}_{j}\left(D\right)\geq r_{\mathrm{th}}$ **then**20:       $P_{\mathrm{cc}}=P_{\mathrm{cc}}+1$;21:   **end if**22:**end for**23:Calculate the simulation value of the ECP, i.e., $P_{a}^{\mathrm{sim}}=P_{\mathrm{ac}}/N$;24:Calculate the simulation value of the percentage of CCA, i.e., $P_{a}^{\mathrm{sim}}=P_{\mathrm{ac}}/N/∆$.

### 5.1. Power Consumption Comparison

**Figure 6:**
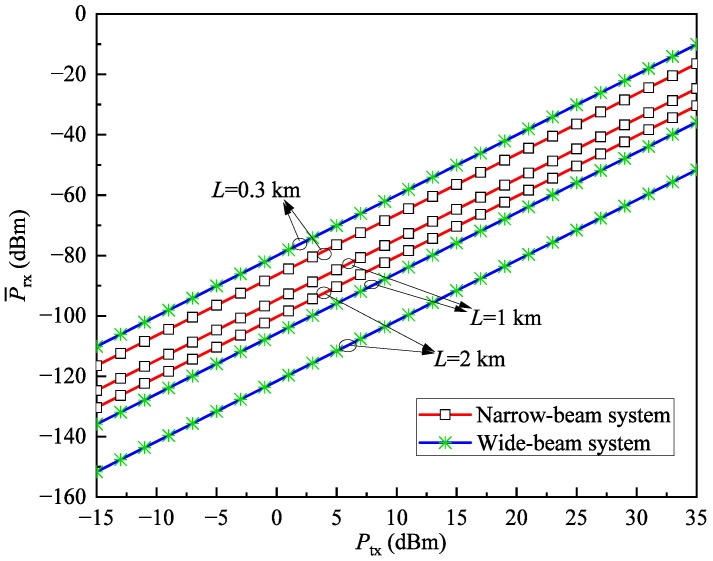
The relationship between the average received power ${\bar{P}}_{\mathrm{rx}}$ and the transmit power $P_{\mathrm{tx}}$ under different transmission distances *L* when V=30km and $r_{\mathrm{th}}=1\;\;\mathrm{dB}$.

To compare the performance of the wide-beam system and the narrow-beam system, Figure 6 illustrates the relationship between the average received power ${\bar{P}}_{\mathrm{rx}}$ and the transmit power $P_{\mathrm{tx}}$ under different transmission distances *L* when V=30km and $r_{\mathrm{th}}=1\;\;\mathrm{dB}$. As can be observed, when *L* is small, the wide-beam system requires lower transmit power to achieve the same received power compared to the narrow-beam system, indicating lower power consumption. However, as *L* increases, the wide-beam system requires higher transmit power to achieve the same received power, leading to higher power consumption. This conclusion is due to the fact that the wide-beam system experiences lower geometric loss at short distances, but its energy dispersion characteristic causes a significant drop in received power at long distances, thus requiring higher transmit power for compensation. In contrast, the narrow-beam system can more effectively concentrate energy at long distances, thereby maintaining lower transmit power demand under the same received power conditions.

### 5.2. Results of ECP

**Figure 7:**
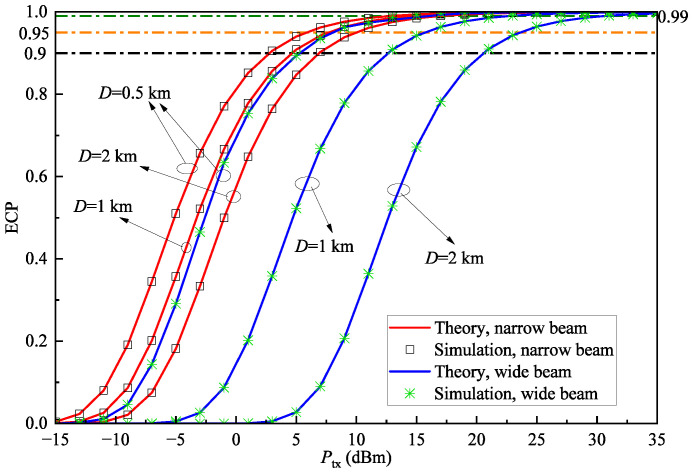
ECP $P_{e}$ versus transmit power $P_{\mathrm{tx}}$ with different cell diameters *D* when $r_{\mathrm{th}}=1\;\;\mathrm{dB}$, and V=30km.

**Figure 8:**
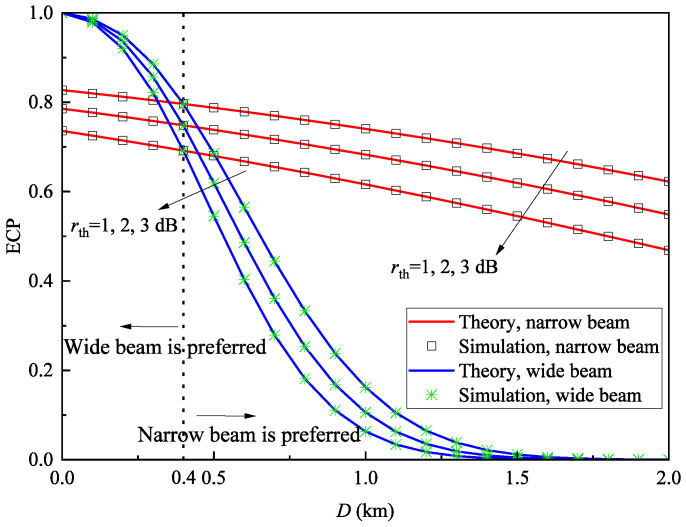
ECP $P_{e}$ versus cell diameter *D* with different SNR thresholds $r_{\mathrm{th}}$ when $P_{\mathrm{tx}}=0\;\;\;\mathrm{dBm}$ and V=30km.

**Figure 9:**
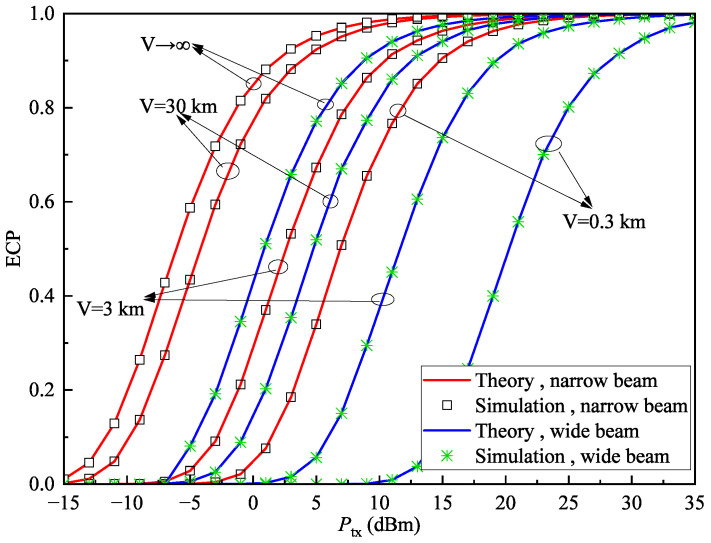
ECP $P_{e}$ versus transmit power $P_{\mathrm{tx}}$ with different visibility conditions *V* when $r_{\mathrm{th}}=1\;\;\mathrm{dB}$ and D=1km.

In this subsection, we present some ECP results, as illustrated in Figure 7, Figure 8 and Figure 9. To facilitate the comparison, the coverage performance of the system is provided for both wide-beam and narrow-beam transmission scenarios at the transmitter.

Since 5G intelligent HSR systems require high quality-of-service, we also plot three ECP levels of 90%, 95%, and 99% [44] for comparisons. As can be observed, all ECP levels can be satisfied by selecting the appropriate transmit power, which verifies that in terms of coverage, the considered system is capable of stably supporting 5G-R. Figure 7 shows the relationship between ECP and transmit power under different cell diameters when $r_{\mathrm{th}}=1\;\;\mathrm{dB}$ and V=30km. The ECP improves significantly with higher transmit power due to SNR enhancement, resulting in better coverage performance. Furthermore, as the cell diameter increases, the ECP decreases. Notably, the wide-beam system shows a significantly lower ECP compared to the narrow-beam system, with a more pronounced rate of decline. This phenomenon mainly stems from two factors: First, longer transmission distances increase atmospheric attenuation, causing more received power loss. Second, compared to the energy-concentrated narrow-beam system, the geometric loss from the energy dispersion in the wide-beam system further reduces received power. This indicates that in deployment scenarios requiring larger coverage areas, narrow-beam configurations should be prioritized to meet quality requirements for edge coverage.

Figure 8 illustrates the relationship between ECP and cell diameter under different SNR thresholds when $P_{\mathrm{tx}}=0\;\;\;\mathrm{dBm}$ and V=30km. Moreover, we present the boundary to indicate when the system uses a wide beam and when it uses a narrow beam. As can be observed, the wide beam is preferred when D<0.4km, while the narrow beam is preferred when D>0.4km. It can be observed that ECP decreases as the SNR threshold increases, which is expected since a higher SNR threshold leads to degraded coverage performance. Additionally, when the cell diameter is small the wide-beam system achieves higher ECP than the narrow-beam system. Within a limited coverage area, the geometric loss caused by energy dispersion in wide-beam systems has a relatively small impact on the received power, whereas narrow-beam systems experience greater power loss due to pointing error. However, as the cell diameter increases, the ECP of the wide-beam system becomes notably lower than that of the narrow-beam system, with a more pronounced decline. The underlying reasons for this behavior are similar to those explained in Figure 7.

Figure 9 presents the relationship between ECP and transmit power under different weather visibility conditions when $r_{\mathrm{th}}=1\;\;\mathrm{dB}$ and D=1km. It can be observed that as visibility decreases, the ECP also declines. Reduced visibility indicates more adverse atmospheric conditions, leading to stronger atmospheric attenuation and thus greater received power loss. The results show that atmospheric changes affect the coverage performance of both wide-beam and narrow-beam systems similarly.

In Figure 7, Figure 8 and Figure 9, the simulation results are in excellent agreement with the theoretical values, with the average relative error of ECP between theoretical and simulation results being only 0.035%, thereby confirming the correctness of the derived ECP expression.

### 5.3. Results of Percentage of CCA

**Figure 10:**
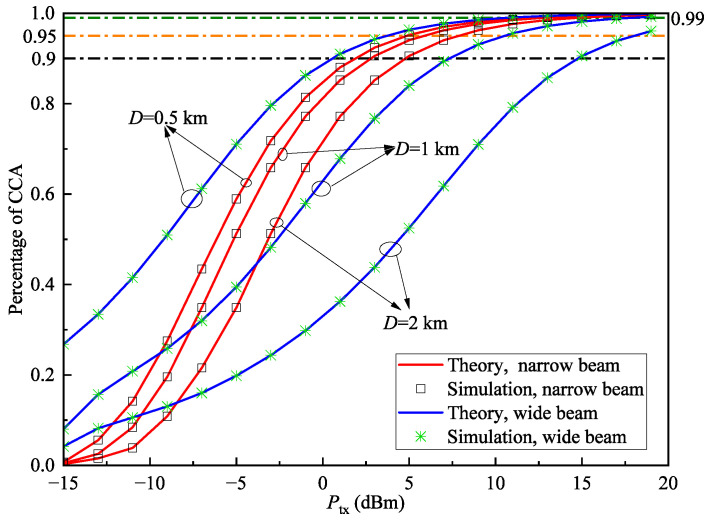
Percentage of CCA $P_{a}$ versus transmit power $P_{\mathrm{tx}}$ with different cell diameters *D* when $r_{\mathrm{th}}=1\;\;\mathrm{dB}$, and V=30km.

**Figure 11:**
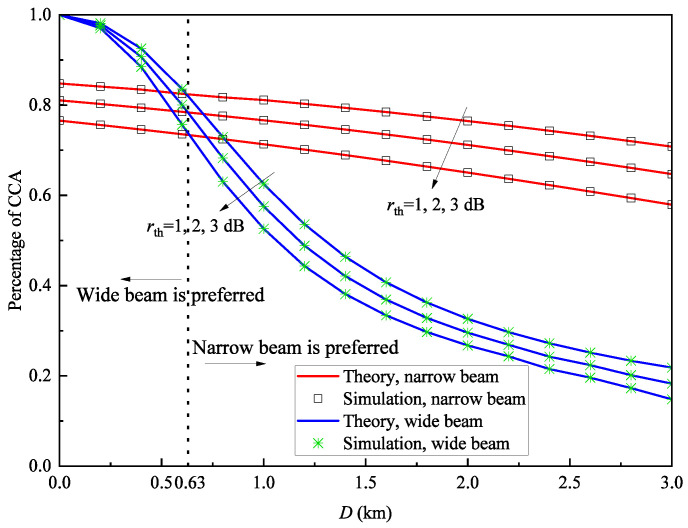
Percentage of CCA $P_{a}$ versus cell diameter *D* with different SNR thresholds $r_{\mathrm{th}}$ when $P_{\mathrm{tx}}=0\;\;\;\mathrm{dBm}$ and V=30km.

**Figure 12:**
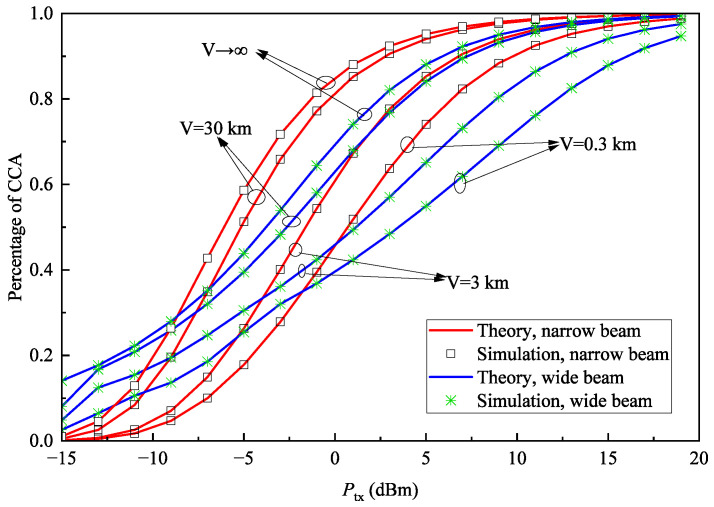
Percentage of CCA $P_{a}$ versus transmit power $P_{\mathrm{tx}}$ with different visibility conditions *V* when $r_{\mathrm{th}}=1\;\;\mathrm{dB}$ and D=1km.

In this section, we present the results for the percentage of CCA in Figure 10, Figure 11 and Figure 12. For comparison, the system’s coverage performance is shown for both wide-beam and narrow-beam transmission modes at the transmitter.

Figure 10 shows the relationship between the percentage of CCA and transmission power under different cell diameters when $r_{\mathrm{th}}=1\;\;\mathrm{dB}$ and V=30km. Similar to Figure 7, all levels of the percentage of CCA can also be satisfied by chosen appropriate transmit power, thus supporting the coverage of the 5G-R. It is clear that the percentage of CCA increases with higher transmit power, as greater power improves the SNR and thus enhances coverage performance. Notably, under the same *D* and $P_{\mathrm{tx}}$, the percentage of CCA consistently exceeds the ECP values, indicating that average cell coverage outperforms edge coverage. As the cell diameter increases, the percentage of CCA gradually decreases. The wide-beam system’s percentage of CCA is not only lower than that of the narrow-beam system but also declines more sharply, consistent with the findings in Figure 7. These results suggest that for large-scale coverage scenarios, narrow-beam configurations should be prioritized to ensure overall coverage quality.

It is particularly noteworthy that at low transmit power levels, the wide-beam system generally achieves a higher percentage of CCA than the narrow-beam system. The wider beam divergence angle of broad beams disperses optical energy over a larger area, enabling the receiver to maintain stable signal reception across an extended coverage range, thereby achieving superior initial coverage performance. However, its lower energy density limits the increase in received signal strength with rising power, resulting in slower growth of the percentage of CCA. In contrast, the narrow beam’s concentrated energy leads to a lower initial percentage of CCA due to stringent pointing accuracy requirements at low power, but its optical power density increases rapidly with transmit power. This enables receivers to obtain a sufficient signal even with minor deviations, achieving faster growth in the percentage of CCA.

Figure 11 shows the relationship between the percentage of CCA and cell radius under different SNR thresholds when $P_{\mathrm{tx}}=0\;\;\;\mathrm{dBm}$ and V=30km. Similar to Figure 8, we also plot the boundary of the cell diameter. As can be observed, the wide beam is preferred when the boundary D<0.63km, while the narrow beam is preferred when D>0.63km. It can be seen that as the SNR threshold increases, the percentage of CCA decreases gradually. Additionally, when the cell diameter is small, the wide-beam system achieves a higher percentage of CCA than the narrow-beam system, for reasons similar to those discussed in Figure 8. Conversely, at larger cell diameters, the wide-beam system’s percentage of CCA falls below that of the narrow-beam system, with a more pronounced decline, consistent with the explanations in Figure 7.

Figure 12 shows the relationship between the percentage of CCA and transmission power under different weather visibility conditions when $r_{\mathrm{th}}=1\;\;\mathrm{dB}$ and D=1km. It can be observed that as visibility decreases, the percentage of CCA also declines. This is because reduced visibility indicates worse atmospheric conditions, leading to an increased atmospheric attenuation coefficient and consequently decreased channel gain. The results demonstrate that atmospheric changes affect the coverage performance of both wide-beam and narrow-beam systems equally. Additionally, at lower transmission power levels, the wide-beam system consistently achieves a higher percentage of CCA than the narrow-beam system. However, as transmission power increases, the narrow-beam system exhibits significantly faster growth in percentage of CCA compared to the wide-beam system, for reasons similar to those explained in Figure 10.

For Figure 10, Figure 11 and Figure 12, the average relative error of the percentage of CCA between theoretical and simulation results is 0.087%; therefore, the gap is small and can be ignored. This verifies the correctness of the derived percentage of CCA expression.

## 6. Conclusions

This paper considers an HSR system based on beamwidth-adaptive FSO communication. Different from previous works, a channel model incorporating both wide and narrow beams is established, and a beamwidth-adaptive method is proposed to enhance the system efficiency. Furthermore, we investigate the coverage performance of the HSR system based on beamwidth-adaptive FSO communication with narrow-strip-shaped cells and the hard handoff scheme. The key contributions of this work are summarized as follows:

- We propose a beamwidth-adaptive FSO communication system and a beamwidth-adaptive method. When the target transmitter emits a wide beam, the system keeps the ATP module off to conserve energy; when a narrow beam is transmitted, the ATP module is activated to ensure precise beam alignment between the transmitter and receiver. This method effectively enables the system to adaptively adjust the state of the ATP module based on the target transmitter’s width during receiver movement, utilizing handover detection and signal power threshold judgment, thereby enhancing communication reliability and energy efficiency.
- For the HSR system based on a beam-adaptive FSO communication system, closed-form expressions of the ECP and the percentage of CCA are derived. All theoretical results match well with simulations, demonstrating the accuracy of the derived expressions for performance evaluation without time-consuming simulations.
- The wide-beam system should be prioritized in small coverage scenarios to ensure coverage quality while saving energy consumption from ATP system activation. The narrow-beam configuration is preferred for large coverage scenarios to meet coverage performance requirements.
- The wide-beam systems perform better in low-power deployment scenarios due to their tolerance to alignment errors, ensuring better initial cell coverage. Narrow-beam systems excel in high-power scenarios, as their energy concentration enables more significant performance improvements with increasing power. System designers should select appropriate beam configurations based on power constraints to optimize the percentage of CCA.

In this paper, we only derive closed-form expressions of the key performance indicators but do not optimize the system parameters. As a future research direction, we will optimize key parameters by the convex optimization theory to further improve system performance. Moreover, the considered HSR system with the straight track can be extended to that with the curved track. In this case, we recommend using smaller cell diameters to account for the receiver moving out of the central part of the wide beam at times or using a narrow beam to eliminate the influence of the curved track.

## Figures and Tables

**Table 1:** Main simulation parameters.

Parameters	Symbols	Values
Photoelectric conversion efficiency	*R*	0.8
Receiver diameter	*B*	0.2 m
Divergence angle	θ	0.01 rad
Noise standard deviation	$\sigma_{n}$	$10^{-6.5}$ A/Hz
Beam wavelength	λ	850 nm
Effective number of large-scale cells	α	3.99
Amount of fading parameter	β	2
Average optical power of the classic scattering component received by off-axis eddies	$\xi_{g}$	0.2
Average optical power of the coherent contributions	$\Omega^{\prime }$	0.5
Beam waist radius	$w_{0}$	$10^{-4}$
Number of sub-intervals in the composite trapezoidal integral	*n*	1000
Number of Snapshots	*N*	$10^{5}$

## Data Availability

The original contributions presented in this study are included in the article.

## Abbreviations

The following abbreviations are used in this manuscript:

5G-R	Fifth generation-railway
ATP	Acquisition-tracking-pointing
BSs	Base stations
CCA	Cell coverage area
ECP	Edge coverage probability
FSO	Free-space optical
GSM-R	Global system for mobile communication-railway
HSR	High-speed railway
LTE-R	Long term evolution-railway
OOK	On-off keying
PDF	Probability density function
RF	Radio frequency
SNR	Signal-to-noise ratio

## Appendix A

**Proof of Theorem** **1.** Substituting (9) into (13), the ECP of the wide-beam system can currently be represented as

(A1) $$\begin{array}{cc} P_{e,1} & =\int_{\gamma_{\mathrm{th}}^{\prime }}^{\infty }A\sum_{k=1}^{\beta }a_{k}{h_{a}}^{\frac{\alpha +k}{2}-1}K_{a-k}\left(2\sqrt{\frac{\alpha \beta h_{a}}{\xi_{g}\beta +\Omega^{\prime }}}\right)dh_{a}\\ \; & =1-\int_{0}^{\gamma_{\mathrm{th}}^{\prime }}A\sum_{k=1}^{\beta }a_{k}{h_{a}}^{\frac{\alpha +k}{2}-1}K_{a-k}\left(2\sqrt{\frac{\alpha \beta h_{a}}{\xi_{g}\beta +\Omega^{\prime }}}\right)dh_{a} \end{array}$$

Changing the order of integral and summation in (A1), we can further obtain

(A2) $$\begin{array}{c} P_{e,1}=1-A\sum_{k=1}^{\beta }a_{k}\int_{0}^{\gamma_{\mathrm{th}}^{\prime }}{h_{a}}^{\frac{\alpha +k}{2}-1}K_{a-k}\left(2\sqrt{\frac{\alpha \beta h_{a}}{\xi_{g}\beta +\Omega^{\prime }}}\right)dh_{a} \end{array}$$

Based on ([45] Equation (24)), which expresses the transformation of the modified Bessel function of the second kind into the Meijer’s G-function, we obtain

(A3) $$\begin{array}{c} P_{e,1}=1-\frac{A}{2}\sum_{k=1}^{\beta }a_{k}\int_{0}^{\gamma_{\mathrm{th}}^{\prime }}{h_{a}}^{\frac{\alpha +k}{2}-1}G_{0,2}^{2,0}\left(\frac{\alpha \beta h_{a}}{\xi_{g}\beta +\Omega^{\prime }}\left|\begin{array}{cc} \frac{\alpha -k}{2} & \frac{k-\alpha }{2} \end{array}\right.\right)dh_{a} \end{array}$$

By ([46] Equation (07.34.21.0084.01)), we obtain (14). □

## Appendix B

**Proof of Theorem** **2.** Substituting (17) into (18), the ECP of the narrow-beam system can currently be represented as

(A4) $$\begin{array}{cc} P_{e,2} & =\int_{\gamma_{\mathrm{th}}^{\prime \prime }}^{\infty }\frac{r^{2}A}{2h}\sum_{k=1}^{\beta }b_{k}G_{1,3}^{3,0}\left(\frac{\alpha \beta e^{\gamma (\lambda )D}h}{\left(\xi_{g}\beta +\Omega^{\prime }\right)A_{0}}\left|\begin{array}{c} 1+r^{2}\\ \begin{array}{ccc} r^{2} & \alpha & k \end{array} \end{array}\right.\right)dh \end{array}$$

Changing the order of integral and summation in (A4), we can further obtain

(A5) $$\begin{array}{c} P_{e,2}=1-\frac{r^{2}A}{2}\int_{0}^{\gamma_{\mathrm{th}}^{\prime \prime }}h^{-1}\sum_{k=1}^{\beta }b_{k}G_{1,3}^{3,0}\left(\frac{\alpha \beta e^{\gamma (\lambda )D}h}{\left(\xi_{g}\beta +\Omega^{\prime }\right)A_{0}}\left|\begin{array}{c} 1+r^{2}\\ \begin{array}{ccc} r^{2} & \alpha & k \end{array} \end{array}\right.\right)dh \end{array}$$

By ([46] Equation (07.34.21.0084.01)), we obtain (19). □

## Appendix C

**Proof of Theorem** **3.** Since the integral calculation of (21) is extremely complex, a composite trapezoidal integral approximation method is adopted here. The calculation formula for the composite trapezoidal rule is given by

(A6) $$\int_{a}^{b}f\left(x\right)\;dx\approx \frac{h}{2}\left[f\left(a\right)+2\sum_{i=1}^{n-1}f\left(x_{i}\right)+f\left(b\right)\right]$$

where the step size can be represented as $h=\frac{b-a}{n}$ and *n* represents the number of subintervals in the composite trapezoidal integral. The partition points are defined as $x_{i}=a+ih$ for i=0,1,…,n. To facilitate (21) by the composite trapezoidal integral approximation method, we define

(A7) $$\begin{array}{cc} f\left(L\right)=\frac{P_{e,1}\left(L\right)}{D} & =\frac{1}{D}-\frac{A}{2D}\sum_{k=1}^{\beta }\left\{a_{k}{\left(\frac{r_{\mathrm{th}}\sigma_{n}\theta^{2}L^{2}e^{\gamma (\lambda )L}}{\sqrt{2}P_{\mathrm{tx}}RB^{2}}\right)}^{\frac{\alpha +k}{2}}\right.\\ \; & \times \left.G_{1,3}^{2,1}\left(\frac{\alpha \beta r_{\mathrm{th}}\sigma_{n}\theta^{2}L^{2}e^{\gamma (\lambda )L}}{\left(\xi_{g}\beta +\Omega^{\prime }\right)\sqrt{2}P_{\mathrm{tx}}RB^{2}}\left|\begin{array}{c} 1-\frac{\alpha +k}{2}\\ \begin{array}{ccc} \frac{\alpha -k}{2} & \frac{k-\alpha }{2} & -\frac{\alpha +k}{2} \end{array} \end{array}\right.\right)\right\} \end{array}$$

According to (A6) and (A7), we can further obtain

(A8) $$P_{a,1}=\int_{0}^{D}f(L)dL=1-\frac{1}{2n}\left(f\left(0\right)+f\left(D\right)+2\sum_{i=1}^{n-1}f\left(\frac{iD}{n}\right)\right)$$

By calculating (A8), we obtain (22). □

## Appendix D

**Proof of Theorem** **4.** Substituting (19) into (24), the percentage of CCA for the narrow-beam system can be expressed as

(A9) $$P_{a,2}=\frac{1}{D}\int_{0}^{D}1-\frac{r^{2}A}{2}\sum_{k=1}^{\beta }b_{k}G_{2,4}^{3,1}\left(\frac{\sigma_{n}\alpha \beta r_{\mathrm{th}}e^{\gamma (\lambda )L}}{\left(\xi_{g}\beta +\Omega^{\prime }\right)\sqrt{2}P_{\mathrm{tx}}RA_{0}}\left|\begin{array}{c} \begin{array}{cc} 1 & 1 \end{array}+r^{2}\\ \begin{array}{cccc} r^{2} & \alpha & k & 0 \end{array} \end{array}\right.\right)dL$$

Changing the order of integral and summation in (A9), we can further obtain

(A10) $$\begin{array}{c} P_{a,2}=1-\frac{r^{2}A}{2}\sum_{k=1}^{\beta }b_{k}\int_{0}^{D}G_{2,4}^{3,1}\left(\frac{\sigma_{n}\alpha \beta r_{\mathrm{th}}e^{\gamma (\lambda )L}}{\left(\xi_{g}\beta +\Omega^{\prime }\right)\sqrt{2}P_{\mathrm{tx}}RA_{0}}\left|\begin{array}{c} \begin{array}{cc} 1 & 1 \end{array}+r^{2}\\ \begin{array}{cccc} r^{2} & \alpha & k & 0 \end{array} \end{array}\right.\right)dL \end{array}$$

Let $t=e^{\gamma (\lambda )L}$, then dL=dt/tγ(λ). Thus, we have

(A11) $$\begin{array}{c} P_{a,2}=1-\frac{r^{2}A\sum_{k=1}^{\beta }b_{k}}{2D\gamma (\lambda )}\int_{1}^{e^{\gamma (\lambda )D}}t^{-1}G_{2,4}^{3,1}\left(\frac{\sigma_{n}\alpha \beta r_{\mathrm{th}}t}{\left(\xi_{g}\beta +\Omega^{\prime }\right)\sqrt{2}P_{\mathrm{tx}}RA_{0}}\left|\begin{array}{c} \begin{array}{cc} 1 & 1 \end{array}+r^{2}\\ \begin{array}{cccc} r^{2} & \alpha & k & 0 \end{array} \end{array}\right.\right)dt \end{array}$$

By ([46] Equation (07.34.21.0003.01)), we can further express $P_{a,2}$ as

(A12) $$\begin{array}{cc} P_{a,2} & =1-\frac{r^{2}A\sum_{k=1}^{\beta }b_{k}}{2D\gamma (\lambda )}G_{3,5}^{3,2}\left(\frac{\sigma_{n}\alpha \beta r_{\mathrm{th}}t}{\left(\xi_{g}\beta +\Omega^{\prime }\right)\sqrt{2}P_{\mathrm{tx}}RA_{0}}\left|\begin{array}{c} \begin{array}{ccc} 1 & 1 & 1+r^{2} \end{array}\\ \begin{array}{cccc} r^{2} & \alpha & k & \begin{array}{cc} 0 & 0 \end{array} \end{array} \end{array}\right.\right)\left|\begin{array}{c} e^{\gamma (\lambda )D}\\ 1 \end{array}\right. \end{array}$$

By calculating (A12), we obtain (25). □

## References

[1] Koide Y., Wakayama N., Maeda T., Watanabe H., Narusue Y., Morikawa H. Propagation characteristics and quality improvement in millimeter-wave communication for ultra-high-speed maglev train. Proceedings of the 27th International Symposium on Wireless Personal Multimedia Communications.

[2] Liu T., Hu F., Ling Z., Li C., Liang Y.-C. (2025). Multi-agent cooperation-based deep reinforcement learning for multisensor perception communication system in HSR tunnel scenario. IEEE Trans. Commun..

[3] Liu X., Chen J., Xie J., Chang Y. (2025). Generating HSR bogie vibration signals via pulse voltage-guided conditional diffusion model. IEEE Trans. Intell. Transp. Syst..

[4] Yan L., Fang X., Fang Y., Hao L., Xue Q., Xu C. (2022). KF-LSTM based beam tracking for UAV-assisted mmWave HSR wireless networks. IEEE Trans. Veh. Technol..

[5] Zheng R., Kong Y., Chan E.H.W., Cao Y., Wang X., Feng X., Guan B. Photonics based microwave frequency shifter for doppler shift compensation in high-speed railways. Proceedings of the Conference on Lasers and Electro-Optics Pacific Rim.

[6] Hu J., Wu W., Wang S., Wu Y., Zhang Z. (2025). Error probability of MPSK with phase errors in dual-hop FSO communication systems. IEEE Photonics Technol. Lett..

[7] Du X., Yu K., Wang Q., Kam P.-Y. (2025). Non-data-aided ML estimation of timing offset and carrier phase for M-APSK modulated FSO systems. IEEE Photonics Technol. Lett..

[8] Songsriboonsit N., Rodrigues T.K., Kawamoto Y., Yamashita Y., Morikawa M., Kato N. (2025). Prediction-based link selection with machine learning in HAP-assisted FSO networks. IEEE Wirel. Commun. Lett..

[9] Mori K., Ishikawa S. Fast handover mechanism for high data rate ground-to-train free-space optical communication system. Proceedings of the IEEE Global Communications Conference.

[10] Weyrauch T., Vorontsov M. (2004). Free-space laser communications with adaptive optics: Atmospheric compensation experiments. J. Opt. Commmun. Rep..

[11] Lee J.H., Shin S., Park G.N., Rhee H.-G., Yang H.-S. (2017). Atmospheric turbulence simulator for adaptive optics evaluation on an optical test bench. Curr. Opt. Photonics.

[12] Galaktionov I., Sheldakova J., Nikitin A., Samarkin V., Parfenov V., Kudryashov A. (2020). Laser beam focusing through a moderately scattering medium using a bimorph mirror. Opt. Exp..

[13] Giggenbach D., Shrestha A. (2022). Atmospheric absorption and scattering impact on optical satellite-ground links. Int. J. Satell. Commun. Netw..

[14] Khuwaja A.A., Zheng G., Chen Y., Feng W. (2019). Optimum deployment of multiple UAVs for coverage area maximization in the presence of co-channel interference. IEEE Access.

[15] Wang X., Turgut E., Gursoy M.C. (2019). Coverage in downlink heterogeneous mmWave cellular networks with user-centric small cell deployment. IEEE Trans. Veh. Technol..

[16] Lin S., Wang H., Li W., Wang J. (2024). Coverage analysis for high-speed railway communications with narrow-strip-shaped cells over Suzuki fading channels. Entropy.

[17] Lin S.-H., Xu Y., Wang J.-Y. (2020). Coverage analysis and optimization for high-speed railway communication systems with narrow-strip-shaped cells. IEEE Trans. Veh. Technol..

[18] Khalighi M.A., Uysal M. (2014). Survey on free space optical communication: A communication theory perspective. IEEE Commun. Surv. Tutor..

[19] Taheri M., Ansari N., Feng J., Rojas-Cessa R., Zhou M. (2017). Provisioning internet access using FSO in high-speed rail networks. IEEE Netw..

[20] Mabrouk W.A., Abdullah M.F.L., Gismalla M.S.M. Enhancement of link range for FSO ground to train communications using multiple transmitters concept. Proceedings of the International Conference on Software Technologies.

[21] Fan Q., Taheri M., Ansari N., Feng J.-H., Rojas-Cessa R., Zhou M.-C., Zhang T.-R. (2018). Reducing the impact of handovers in ground-to-train free space optical communications. IEEE Trans. Veh. Technol..

[22] Lin S.-H., Xu Y., Wang L., Wang J.-Y. (2022). Coverage analysis and chance-constrained optimization for HSR communications with carrier aggregation. IEEE Trans. Intel. Transport. Syst..

[23] Zhang J., Du H., Zhang P., Cheng J., Yang L. (2020). Performance analysis of 5G mobile relay systems for high-speed trains. IEEE J. Sel. Areas Commun..

[24] Nace D., Pióro M., Poss M., D’Andreagiovanni F., Kalesnikau I., Shehaj M., Tomaszewski A. (2019). An optimization model for robust FSO network dimensioning. Opt. Switch. Netw..

[25] Farid A.A., Hranilovic S. Optimization of beam width, bit error rate and availability for free-space optical links. Proceedings of the 6th International Symposium on Communication Systems, Networks and Digital Signal Processing (CSNDSP).

[26] Mohammad A.B. Optimization of FSO system in tropical weather using multiple beams. Proceedings of the IEEE 5th International Conference on Photonics (ICP).

[27] Bloom S., Korevaar E., Schuster J., Willebrand H. (2003). Understanding the performance of free-space optics [invited]. J. Opt. Netw..

[28] Kaymak Y., Rojas-Cessa R., Feng J., Ansari N., Zhou M. (2017). On divergence-angle efficiency of a laser beam in free-space optical communications for high-speed trains. IEEE Trans. Veh. Technol..

[29] Killinger D. (2002). Free space optics for laser communication through the air. Opt. Photonics News..

[30] Wang J.-Y., Wang J.-B., Chen M., Tang Y., Zhang Y. (2014). Outage analysis for relay-aided free-space optical communications over turbulence channels with nonzero boresight pointing errors. IEEE Photonics J..

[31] He J., Norwood R.A., Brandt-Pearce M., Djordjevic I.B., Cvijetic M., Subramaniam S., Himmelhuber R., Reynolds C., Blanche P., Lynn B. (2014). A survey on recent advances in optical communications. Comput. Electr. Eng..

[32] Turan H., Subaşi Ö. Development of fine tracking unit for hybrid ATP mechanism in free-space optical communication. Proceedings of the 29th Signal Processing and Communications Applications Conference.

[33] Mai V., Kim H. (2022). Beam steering and divergence control using variable focus liquid lenses for WDM FSO communications. IEEE Photonics Technol. Lett..

[34] Paudel R., Ghassemlooy Z., Le-Minh H., Rajbhandari S. (2013). Modelling of free space optical link for ground-to-train communications using a gaussian source. IET Optoelectron..

[35] Fan Q., Ansari N., Feng J., Rojas-Cessa R., Zhou M., Zhang T. (2018). Reducing the number of FSO base stations with dual transceivers for next-generation ground-to-train communications. IEEE Trans. Veh. Technol..

[36] Lv L., Yang Z., Fang Y., Guizani M. (2024). Adaptive interleaver and rate-compatible PLDPC code design for MIMO FSO-RF systems. IEEE Trans. Veh. Technol..

[37] Rakia T., Yang H.-C., Alouini M.-S., Gebali F. (2015). Outage analysis of practical FSO/RF system with adaptive combining. IEEE Commun. Lett..

[38] Xu G., Xu M., Zhang Q., Song Z. (2024). Cooperative FSO/RF space-air-ground integrated network system with adaptive combining: A performance analysis. IEEE Trans. Wirel. Commun..

[39] Ansari I.S., Yilmaz F., Alouini M.-S. (2016). Performance analysis of free-space optical links over Málaga (M) turbulence channels with pointing errors. IEEE Trans. Wirel. Commun..

[40] Khatib M. (2014). Contemporary Issues in Wireless Communications.

[41] Navas A.J., Balsells J.M.G., Paris J.F., Vazquez M.C., Notario A.P. (2012). Impact of pointing errors on the performance of generalized atmospheric optical channels. Opt. Express.

[42] Elsawy Y., Alatawi A.S., Abaza M., Moawad A., Agoune E.-H. (2024). Next-generation dual transceiver FSO communication system for high-speed trains in neom smart city. Photonics.

[43] (2007). Safety of Laser Products-Part 1: Equipment Classification and Requirements.

[44] Ai B., He R., Li G., Guan K., He D., Shi G., Zhong Z. (2017). Determination of cell coverage area and its applications in high-speed railway environments. IEEE Trans. Veh. Technol..

[45] Adamchik V.S., Marichev O.I. The algorithm for calculating integrals of hypergeometric type functions and its realization in REDUCE system. Proceedings of the International Symposium on Symbolic and Algebraic Computation (ISSAC).

[46] Wolfram The Wolfram Functions Site. http://functions.wolfram.com/.

